# Percutaneous retrograde revascularization of chronic occlusions of the superior mesenteric artery via collaterals of the celiac artery

**DOI:** 10.1186/s42155-020-00170-x

**Published:** 2020-11-14

**Authors:** Wilhelm H. Kersjes, Alexander Hesse

**Affiliations:** Institute of Diagnostic and Interventional Radiology, Klinikum Ludwigsburg, Posilipostr. 4, 71640 Ludwigsburg, Germany

**Keywords:** Chronic total occlusion, Chronic mesenteric ischemia, Superior mesenteric artery, Celiac artery, Percutaneous transluminal angioplasty, Stent, Collaterals, Technique

## Abstract

**Purpose:**

To evaluate the technical success of percutaneous retrograde revascularization of the superior mesenteric artery (SMA) via the celiac artery (CA) in patients with chronic mesenteric ischemia (CMI).

**Methods:**

We performed a retrospective review of three patients with chronic total occlusions (CTOs) of the origin of SMA which were recanalized retrograde via collaterals of the CA after frustrating attempt of antegrade revascularization from the abdominal aorta in our institute between May 2019 and June 2020.

**Results:**

All technical procedures of retrograde revascularization of CTOs of SMA via collaterals of the CA were successful. The clinical outcome resulted in a sustained resolution of abdominal pain in all cases.

**Conclusion:**

Retrograde recanalization of SMA via collaterals from the CA seems to be a successful endovascular option for patients with CMI and a chronically occluded superior mesenteric artery when antegrade recanalization fails as far as it can be concluded from the small number of presented cases.

## Background

Abdominal angina often manifests as a symptom of severe vascular disease with involvement of the SMA and / or the CA. Endovascular procedures have been increasingly used to treat CMI. Due to the mostly existing concomitant diseases and the reduced general condition of the underlying patient population, endovascular treatment appears safer than open surgery and it is characterized by its high technical and clinical success rates and its low complication rates (Landis et al. 2005).

However, CTOs of the SMA continue to pose a particular challenge in the case of endovascular therapy of CMI. In individual reports the results of endovascular recanalization in patients with chronic occlusions of the SMA and the CA are discussed (Sharafuddin et al. 2012; Grilli et al. 2014). If the endovascular antegrade revascularization of the SMA is unsuccessful and open surgical interventions are associated with higher morbidity and mortality due to existing comorbidities, a further option is given by a subsequent retrograde revascularization of the SMA via collaterals from the CA as shown in this collective. There are only a few case reports in the literature using this technique (Grilli et al. 2014; Stephens et al. 2010; Robken and Shammas 2007).

The purpose of the present study is to evaluate the technique of retrograde recanalization of the SMA via collaterals from the CA.

## Patients

In the time period between May 2019 and June 2020 in our institute three consecutive patients with CMI and CTOs of the SMA have been recanalized and stented in a retrograde manner via collaterals from the CA.

In two of these patients we found an additional stenosis of the CA. These stenoses were previously dilated and stented. Because the symptoms didn’t improve adequately after therapy, in a further intervention the frustrating attempt to recanalize the chronically occluded SMA directly from the abdominal aorta was performed. After that the recanalization of the proximally occluded SMA was successful using retrograde passage over collaterals from the CA in the same session.

In the third patient, after a failed attempt to recanalize the occluded SMA antegradely, retrograde recanalization of the occlusion of the SMA was done via collaterals from the CA in a second examination.

The patient population consisted of three women aged 77, 87 and 89 years. The complained symptoms had existed in all for several months, consisting of postprandial pain (3x), weight loss (3x; 10–15 kg) and diarrhea (1x).

Cardiovascular risk factors included hypertension (3x), hyperlipidemia (2x) and coronary heart disease (1x).

### Technical procedure

Duplex ultrasonography was performed in all cases as pre-interventional imaging. Additionally abdominal computed tomography (CT) - and magnetic resonance imaging (MRI) – examinations were carried out in 2 respectively 1 case.

**Fig. 1:**
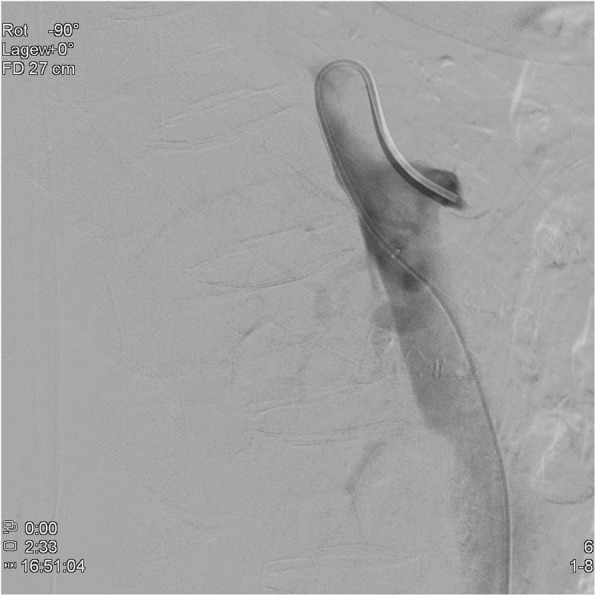
Abdominal angiography in lateral projection with a Sidewinder 1-catheter in the stump of chronically occluded SMA. Attempt of antegrade recanalization was not successful

**Fig. 2:**
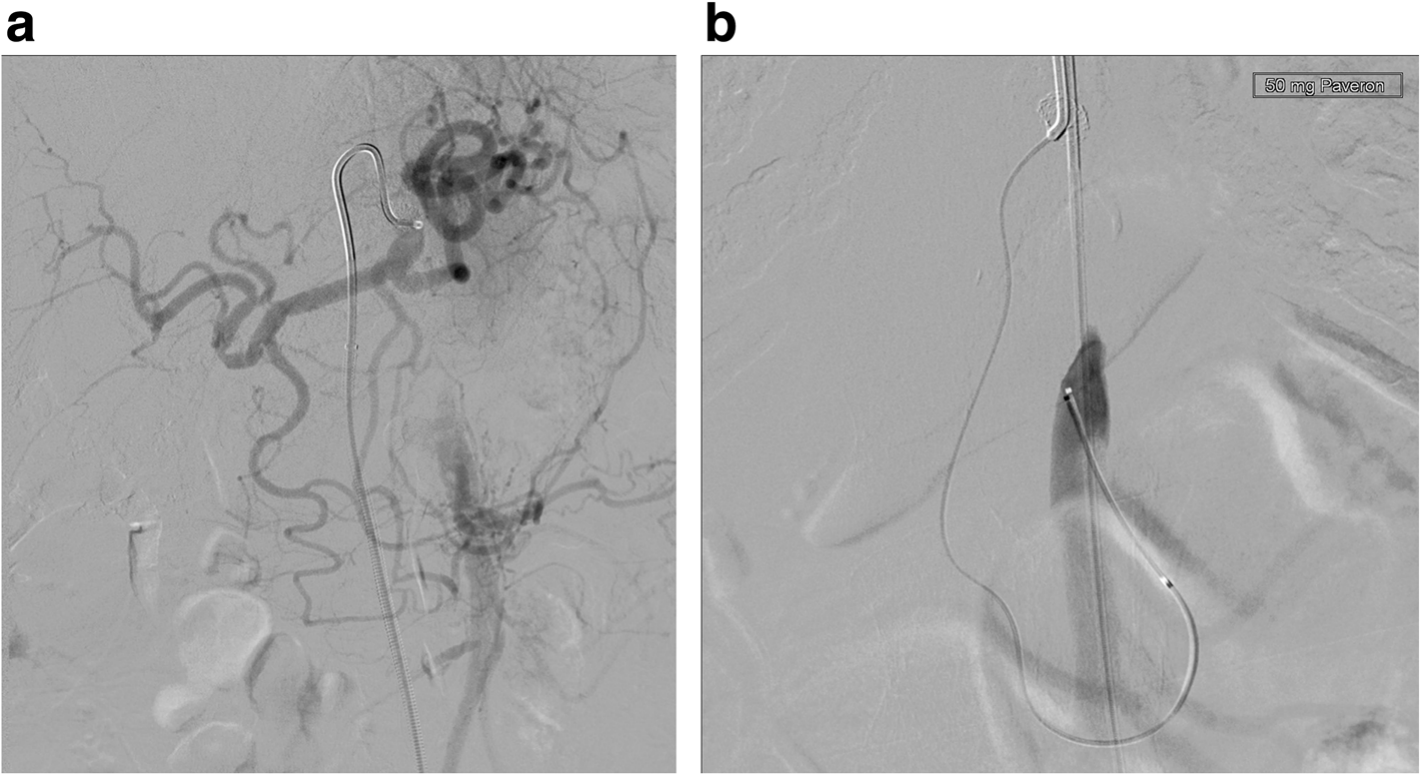
Same patient as in Fig. 1. **a** Selective angiography of the celiac artery with a Sidewinder I-catheter. **b** After passage of the gastroduodenal artery and the pancreaticoduodenal arcade with a coaxially inserted microcatheter the SMA is retrogradely filled up to the proximal occlusion

**Fig. 3:**
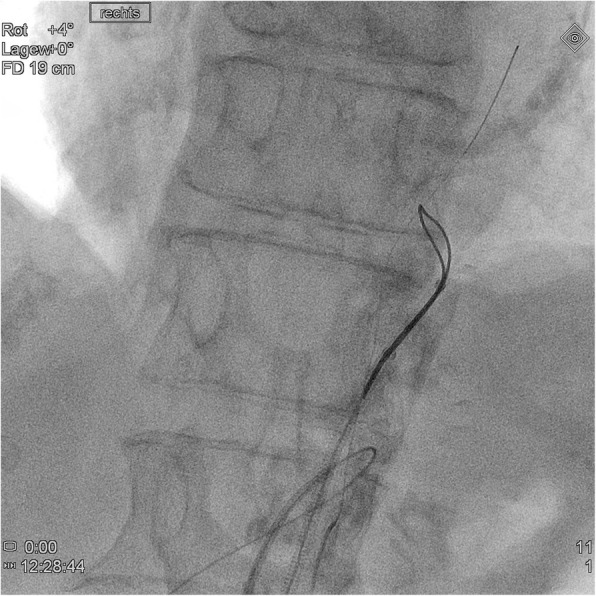
The free end of the 0.014-in. guide wire was captured with a loop catheter and externalized through the sheath in the left common femoral artery

**Fig. 4:**
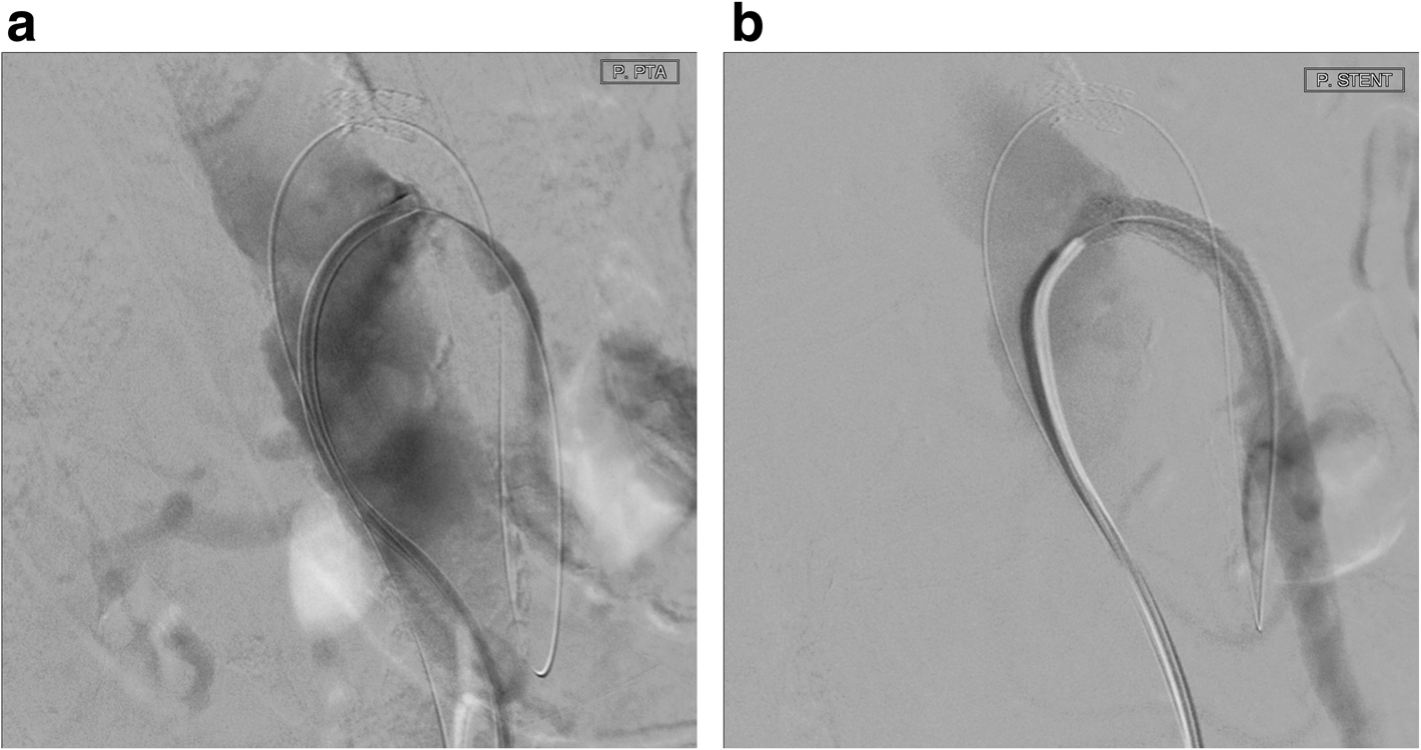
Lateral projection of abdominal aorta: The occlusion of the SMA (**a**) was supplied with 2 overlapping stents (**b**) via the left femoral artery access

In all cases, a long 5-French (F) sheath was placed in the right common femoral artery percutaneously. Abdominal angiography with a pigtail catheter in several acquisition planes, including a lateral projection, was followed by selective angiography (Sidewinder I or Cobra catheter) of the SMA (Fig. 1). With a 0.018- or 0.014-in. guide wire, the attempt was made to recanalize the occlusion directly from the abdominal aorta. This attempt was frustrating in all cases. Therefore, the celiac artery was selectively probed with a Cobra- or Sidewinder I-catheter. With a coaxially inserted microcatheter and a 0.014-in. guide wire (300 mm), the passage was then made through the gastroduodenal artery and further via pancreatic arcades into the SMA (Fig. 2). In all cases, the occlusion located proximally in the SMA was first passed with the guide wire and then with the microcatheter from retrograde. The intraluminal position of the tip of the microcatheter was checked by contrast injection. After successful retrograde recanalization of the occlusion of the SMA with the guide wire and the microcatheter, an additional long 5-F sheath was introduced into the left groin. The free end of the 0.014-in. guide wire was captured with a loop catheter inserted through the sheath in the left common femoral artery and externalized (Fig. 3). The microcatheter was then carefully removed over the right sheath via the collaterals of CA. A thin-lumen balloon catheter was thereafter passed over the 0.014-in. guide wire through the left groin to the occluded segment of the SMA and this section was carefully dilated. In all cases we started with a 3 mm × 40 mm balloon; in 2 of the interventions a 4 mm × 40 mm balloon was additionally used afterwards. After removing the balloon catheter, the former occlusion of the SMA was supplied in all cases with a 5 mm × 18 mm balloon-expandable stent pre-mounted on a balloon at the distal tip of a rapid exchange-type delivery catheter. In one patient a second overlapping stent of the same type and size was implanted also via the left femoral artery access (Fig. 4). The catheters and sheaths were finally removed and the access routes supplied with closure systems.

## Results

In all three patients the technical procedure of recanalization was successful using the retrograde passage via collaterals of the CA. The symptoms which caused the treatment disappeared completely so that they were symptom-free immediately after intervention. We didn’t notice any complications caused by the interventions.

**Fig. 5:**
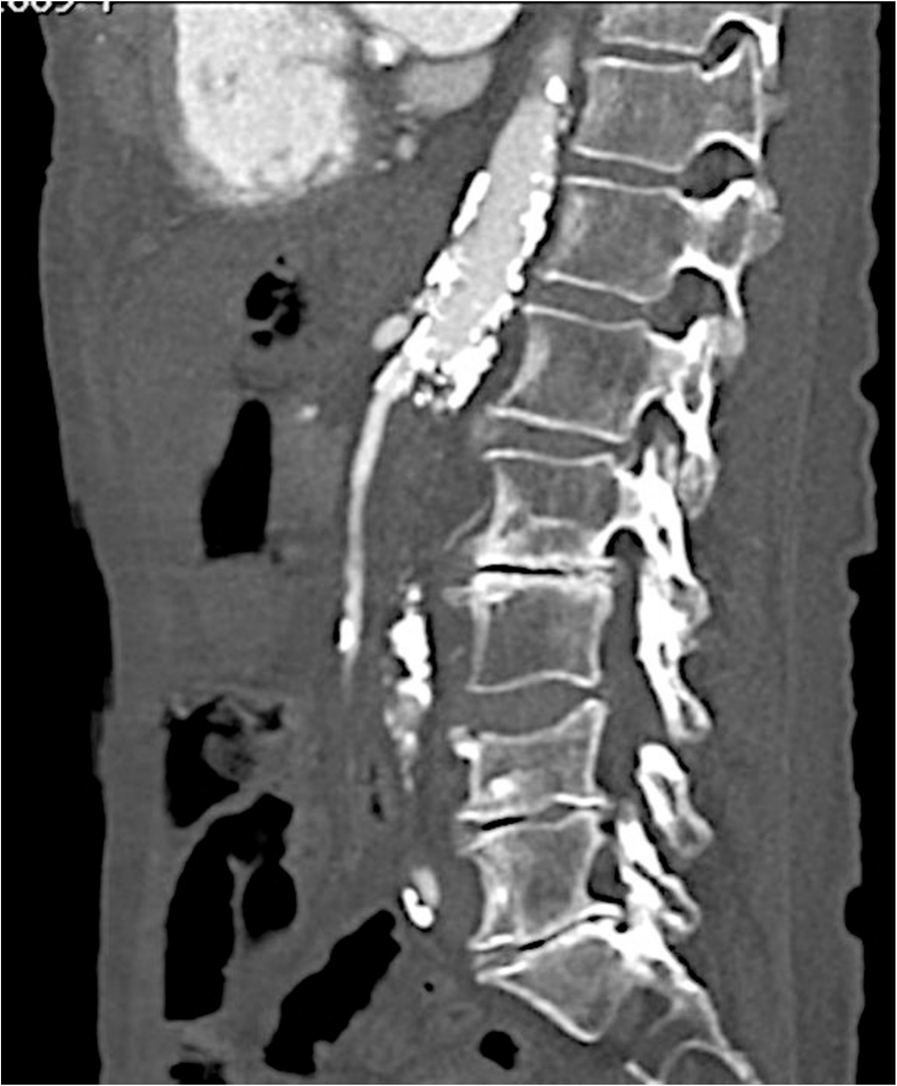
Follow-up CT of the abdomen 13 months after revascularization and stenting of the SMA with free perfusion of the vessel

Follow-up results proved that all patients are still alive and that they all had sustained resolution of abdominal pain for 3, 12 and 13 months. One of the patients got a follow-up CT of the abdomen 13 months after the revascularization which revealed a continuous perfusion of the stented SMA (Fig. 5).

## Discussion

The percutaneous treatment of stenoses of the SMA and the CA in patients with CMI is now accepted as a less invasive standard therapy compared to the operative therapy approach although surgical revascularizations in CMI show a superior long-term patency and minimal need for secondary procedures. Surgical revascularization is effective, but is associated with an increased risk of morbidity and mortality in the mostly malnourished and multimorbid patients.

The occluded SMA, however, remains a challenge for the percutaneous therapeutic approach. Individual papers report high technical and clinical success rates with low complication rates in connection with the antegrade reopening of CTOs of the SMA (Sharafuddin et al. 2012; Grilli et al. 2014; Sarac et al. 2008; Chahid et al. 2004). But subintimal recanalization can be problematic because it can be associated with the risk of uncontrolled antegrade dissection with occlusion of side branches of the SMA. In addition, since the location of the reentry point is more or less random, this results in the need for longer and more stents. By that the rate of restenosis and the risk of occlusion of side branches is increased. Therefore, subintimal recanalization should be avoided if possible (Grilli et al. 2014).

If an occluded SMA cannot be recanalized antegradely from the abdominal aorta, the only alternative besides surgical revascularization is a percutaneous retrograde attempt to recanalize via collaterals of the CA. The results of the present report show that after a failed attempt of endovascular antegrade revascularization of the SMA, a retrograde revascularization of the proximal occlusion of the SMA via collaterals from the CA is very promising. But the success rate of the described technique has to be verified by further examinations.

The complication and success rates of brachial versus femoral approach are controversially discussed in the literature. All our interventions were done via femoral access. Alternatively, a bilateral or a brachial and a femoral approach would of course have been possible. The choice of the access route(s) depends on the examiner’s preference and the anatomical conditions of the patient. If a visible stump of the superior mesenteric artery is directed obliquely downwards, the inserted catheter is often more stable after brachial access and supports recanalization. Most authors describe brachial access as superior to femoral access (Sarac et al. 2008; Peck et al. 2010).

In a previous report of retrograde recanalization of SMA the authors used an 8-F sheath, 8-F guiding catheters and 0.035-in. guide wires (Robken and Shammas 2007). In patients with small-vessel conditions, particularly the collaterals between the CA and SMA may develop vascular spasms or other mechanical problems that hinder success. In the cases described here, 5-F sheaths and small microcatheters with 0.014-in. guide wires were used, with which the collaterals could be passed easily.

## Conclusions

Retrograde recanalization of the superior mesenteric artery via collaterals from the CA is a newer endovascular option for patients with CMI and a chronically occluded SMA when antegrade recanalization fails as far as it can be concluded from the small number of patients. Especially in elderly patients endovascular approaches are feasible, but technically challenging options to treat patients with CMI because of high risk of perioperative complications.

## Data Availability

Not applicable.

## Abbreviations

CA: Celiac artery
CMI: Chronic mesenteric ischemia
CT: Computed tomography
CTO: Chronic total occlusion
F: French
Fig: Figure
MRI: Magnetic resonance imaging
SMA: Superior mesenteric artery
